# Generalized Bifuzzy Lie Subalgebras

**DOI:** 10.1155/2013/365065

**Published:** 2013-12-30

**Authors:** Noura Alshehri, Muhammad Akram

**Affiliations:** ^1^Department of Mathematics, Faculty of Sciences (Girls), King Abdulaziz University, Jeddah, Saudi Arabia; ^2^Punjab University College of Information Technology, University of the Punjab, Old Campus, Lahore, Pakistan

## Abstract

We introduce the concept of (*γ*, *δ*)-bifuzzy Lie subalgebra, where *γ*, *δ* are any two of {∈, *q*, ∈∨*q*, ∈∧*q*} with *γ* ≠ ∈∧*q*, by using belongs to relation (∈) and quasi-coincidence with relation (*q*) between bifuzzy points and bifuzzy sets and discuss some of its properties. Then we introduce bifuzzy soft Lie subalgebras and investigate some of their properties.

## 1. Introduction

The concept of Lie groups was first introduced by Sophus Lie in nineteenth century through his studies in geometry and integration methods for differential equations. Lie algebras were also discovered by him when he attempted to classify certain smooth subgroups of a general linear group. The importance of Lie algebras in mathematics and physics has become increasingly evident in recent years. In applied mathematics, Lie theory remains a powerful tool for studying differential equations, special functions, and perturbation theory. It is noted that Lie theory has applications not only in mathematics and physics but also in diverse fields such as continuum mechanics, cosmology, and life sciences. A Lie algebra has nowadays even been applied by electrical engineers in solving problems in mobile robot control [1].

After introducing the concept of fuzzy sets by Zadeh [2] in 1965, there are many generalizations of this fundamental concept. Among these new concepts, the concept of intuitionistic fuzzy sets given by Atanassov [3] in 1983 is the most important and interesting one because it is simply an extension of fuzzy sets. In 1995, Gerstenkorn and Mańko [4] renamed the intuitionistic fuzzy sets as *bifuzzy sets*. The elements of the bifuzzy sets are featured by an additional degree which is called the degree of uncertainty. This kind of fuzzy sets have now gained a wide recognition as a useful tool in the modeling of some uncertain phenomena. Bifuzzy sets have drawn the attention of many researchers in the last decades. This is mainly due to the fact that bifuzzy sets are consistent with human behavior, by reflecting and modeling the hesitancy present in real-life situations. In fact, the fuzzy sets give the degree of membership of an element in a given set, while bifuzzy sets give both a degree of membership and a degree of nonmembership. As for fuzzy sets, the degree of membership is a real number between 0 and 1. This is also the case for the degree of nonmembership, and furthermore the sum of these two degrees is not greater than 1.

In 1999, Molodtsov [5] initiated the novel concept of soft set theory to deal with uncertainties which can not be handled by traditional mathematical tools. He successfully applied the soft set theory several disciplines, such as game theory, Riemann integration, Perron integration, and measure theory. Applications of soft set theory in real life problems are now catching momentum due to the general nature parametrization expressed by a soft set. Maji et al. [6] gave first practical application of soft sets in decision making problems. They also presented the definition of intuitionistic fuzzy soft set [7]. Yehia introduced the notion of fuzzy Lie subalgebras of Lie algebras in [8] and studied some results. Akram and Feng introduced the notion of soft Lie subalgebras of Lie algebras in [9] and studied some of their results. Akram et al. introduced the notions of fuzzy soft Lie subalgebras in [10]. In this paper, we introduce a new kind of generalized bifuzzy Lie algebra, an (*γ*, *δ*)-bifuzzy Lie algebra, and discuss some of its properties. Then we introduce bifuzzy soft Lie algebras and investigate some of their properties. We use standard definitions and terminologies in this paper.

## 2. Preliminaries

In this section, we review some known basic concepts that are necessary for this paper.

A *Lie algebra* is a vector space *L* over a field *F* (equal to **R** or **C**) on which *L* × *L* → *L* denoted by (*x*, *y*)→[*x*, *y*] is defined satisfying the following axioms:

(L1) [*x*, *y*] is bilinear,
(L2) [*x*, *x*] = 0 for all *x* ∈ *L*,
(L3) [[*x*, *y*], *z*] + [[*y*, *z*], *x*] + [[*z*, *x*], *y*] = 0 for all *x*, *y*, *z* ∈ *L* (Jacobi identity).

Throughout this paper, *L* is a Lie algebra and *F* is a field. We note that the multiplication in a Lie algebra is not associative; that is, it is not true in general that [[*x*, *y*], *z*] = [*x*, [*y*, *z*]]. But it is *anticommutative*; that is, [*x*, *y*] = −[*y*, *x*]. A subspace *H* of *L* closed under [·, ·] will be called a *Lie subalgebra*.

Let *μ* be a *fuzzy set* on *X*; that is, a map *μ* : *X* → [0,1]. As an important generalization of the notion of fuzzy sets in *X*, Atanassov introduced the concept of a *bifuzzy set* defined on a nonempty set *X* as objects having the form

(1) $$\begin{array}{c} A=\left(\mu_{A},\nu_{A}\right)=\left\{\left(x,\mu_{A}\left(x\right),\nu_{A}\left(x\right)\right)\;|\;x\in X\right\}, \end{array}$$

where the functions *μ* : *X* → [0,1] and *ν* : *X* → [0,1] denote the *degree of membership* (viz., *μ*_*A*_(*x*)) and the *degree of nonmembership* (viz., *ν*_*A*_(*x*)) of each element *x* ∈ *X* to the set *A*, respectively, and 0 ≤ *μ*_*A*_(*x*) + *ν*_*A*_(*x*) ≤ 1 for all *x* ∈ *X*.

**Definition 1 (see [3, 11])**

Let *A* = (*μ*_*A*_, *ν*_*A*_) be a bifuzzy set on *X* and let *s*, *t* ∈ [0,1] be such that *s* + *t* ≤ 1. Then the set

(2) $$\begin{array}{c} A_{\left(s,t\right)}=\left\{x\;|\;\mu_{A}\left(x\right)\geq s,\nu_{A}\left(x\right)\leq t\right\} \end{array}$$

is called an (*s*, *t*)*-level subset* of *A*. *A*_(*s*,*t*)_ is a crisp set.

**Definition 2 (see [12])**

A bifuzzy set *A* = (*μ*_*A*_, *ν*_*A*_) on *L* is called a *bifuzzy Lie subalgebra* if the following conditions are satisfied:

- *μ*_*A*_(*x* + *y*) ≥ min⁡{*μ*_*A*_(*x*), *μ*_*A*_(*y*)},
- *ν*_*A*_(*x* + *y*) ≤ max⁡{*ν*_*A*_(*x*), *ν*_*A*_(*y*)},
- *μ*_*A*_(*αx*) ≥ *μ*_*A*_(*x*), *ν*_*A*_(*αx*) ≤ *ν*_*A*_(*x*),
- *μ*([*x*, *y*]) ≥ max⁡{*μ*(*x*), *μ*(*y*)},
- *ν*([*x*, *y*]) ≤ min⁡{*ν*(*x*), *ν*(*y*)}

for all *x*, *y* ∈ *L* and *α* ∈ *F*.

**Definition 3 (see [13])**

Let *c* be a point in a nonempty set *X*. If *γ* ∈ (0,1] and *δ* ∈ [0,1) are two real numbers such that 0 ≤ *γ* + *δ* ≤ 1, then the bifuzzy set *c*(*γ*, *δ*) = 〈*x*, *c*_*γ*_, 1 − *c*_1−*δ*_〉 is called a bifuzzy point (BP for short) in *X*, where *γ* (resp., *δ*) is the degree of membership (resp., nonmembership) of *c*(*γ*, *δ*) and *c* ∈ *X* is the support of *c*(*γ*, *δ*). Let *c*(*γ*, *δ*) be a BP in *X* and let *A* = 〈*x*, *μ*_*A*_, *ν*_*A*_〉 be a bifuzzy set in *X*. Then *c*(*γ*, *δ*) is said to belong to *A*, written *c*(*γ*, *δ*) ∈ *A*, if *μ*_*A*_(*c*) ≥ *γ* and *ν*_*A*_(*c*) ≤ *δ*. We say that *c*(*γ*, *δ*) is quasicoincident with *A*, written *c*(*γ*, *δ*)*qA*, if *μ*_*A*_(*c*) + *γ* > 1 and *ν*_*A*_(*c*) + *δ* < 1. To say that *c*(*γ*, *δ*)∈∨*qA* (resp., *c*(*γ*, *δ*)∈∧*qA*) means that *c*(*γ*, *δ*) ∈ *A* or *c*(*γ*, *δ*)*qA* (resp., *c*(*γ*, *δ*) ∈ *A* and *c*(*γ*, *δ*)*qA*) and $c(\gamma ,\delta )\bar{\in \vee q}A$ means that *c*(*γ*, *δ*)∈∨*qA* does not hold.

**Definition 4**

Let *f* and *g* be any two bifuzzy subsets of *L*. Then the sum *f* + *g* is a bifuzzy subset of *L* defined by

(3) (μf+μg)(z) ={⋁z=[x,y]⁡(μf(a)∧μf(b)∧μg(a)∧μg(b))for z=[x,y],0otherwise,(νf+νg)(z) ={⋀z=[x,y]⁡(νf(a)∨νf(b)∨νg(a)∨νg(b))for z=[x,y],1otherwise.

Let IF(*U*) denote the family of all bifuzzy sets in *U*.

**Definition 5 (see [7])**

Let *U* be an initial universe and *A*⊆*E* a set of parameters. A pair (*f*, *A*) is called a *bifuzzy soft set* over *U*, where *f* is a mapping given by *f* : *A* → *IF*(*U*). A bifuzzy soft set is a parameterized family of bifuzzy subsets of *U*. For any *ɛ* ∈ *A*, *f*_*ɛ*_ is referred to as the set of *ɛ*-approximate elements of the bifuzzy soft set (*f*, *A*), which is actually a bifuzzy set on *U* and can be

(4) $$\begin{array}{c} f_{ɛ}=\left\{\left(\mu_{f_{ɛ}}\left(x\right),\nu_{f_{ɛ}}\left(x\right)\right)\;|\;x\in U\right\}, \end{array}$$

where *μ*_*f*_*ɛ*__(*x*) and *ν*_*f*_*ɛ*__(*x*) are the membership degree and nonmembership degree that object *x* holds on parameter *ɛ*, respectively.

**Definition 6 (see [7])**

Let (*f*, *A*) and (*g*, *B*) be two bifuzzy soft sets over *U*. We say that (*f*, *A*) is a *bifuzzy soft subset* of (*g*, *B*) and write (*f*, *A*)⋐(*g*, *B*) if

(i) *A*⊆*B*,
(ii) for any *ɛ* ∈ *A*, *f*(*ɛ*)⊆*g*(*ɛ*).

(*f*, *A*) and (*g*, *B*) are said to be bifuzzy soft equal and write (*f*, *A*) = (*g*, *B*) if (*f*, *A*)⋐(*g*, *B*) and (*g*, *B*)⋐(*f*, *A*).

**Definition 7 (see [7, 14])**

Let (*f*, *A*) and (*g*, *B*) be two bifuzzy soft sets over *U*. Then their *extended intersection* is a bifuzzy soft set denoted by (*h*, *C*), where *C* = *A* ∪ *B* and

(5) $$\begin{array}{c} h\left(ɛ\right)=\{\begin{array}{cc} f_{ɛ} & \text{if}\;ɛ\in A-B,\\ g_{ɛ} & \text{if}\;ɛ\in B-A,\\ f_{ɛ}\cap g_{ɛ} & \text{if}\;ɛ\in A\cap B, \end{array} \end{array}$$

for all *ɛ* ∈ *C*. This is denoted by $\left(h,C)=(f,A)\;\tilde{\cap }\;(g,B\right)$.

**Definition 8 (see [7, 14])**

If (*f*, *A*) and (*g*, *B*) are two bifuzzy soft sets over the same universe *U* then “(*f*, *A*) AND (*g*, *B*)” is a bifuzzy soft set denoted by (*f*, *A*)∧(*g*, *B*) and is defined by (*f*, *A*)∧(*g*, *B*) = (*h*, *A* × *B*), where, *h*(*a*, *b*) = *h*(*a*)∩*g*(*b*) for all (*a*, *b*) ∈ *A* × *B*. Here ∩ is the operation of a bifuzzy intersection.

**Definition 9 (see [7, 14])**

Let (*f*, *A*) and (*g*, *B*) be two bifuzzy soft sets over *U*. Then their *extended union* is denoted by (*h*, *C*), where *C* = *A* ∪ *B* and

(6) $$\begin{array}{c} h\left(ɛ\right)=\{\begin{array}{cc} f_{ɛ} & \text{if}\;ɛ\in A-B,\\ g_{ɛ} & \text{if}\;ɛ\in B-A,\\ f_{ɛ}\cup g_{ɛ} & \text{if}\;ɛ\in A\cap B, \end{array} \end{array}$$

for all *ɛ* ∈ *C*. This is denoted by $\left(h,C)=(f,A)\;\tilde{\cup }\;(g,B\right)$.

**Definition 10 (see [7, 14])**

Let (*f*, *A*) and (*g*, *B*) be two fuzzy soft sets over a common universe *U* with *A*∩*B* ≠ *∅*. Then their *restricted intersection* is a bifuzzy soft set (*h*, *A*∩*B*) denoted by (*f*, *A*)⋓(*g*, *B*) = (*h*, *A*∩*B*), where *h*(*ɛ*) = *f*(*ɛ*)∩*g*(*ɛ*) for all *ɛ* ∈ *A*∩*B*.

**Definition 11 (see [7, 14])**

Let (*f*, *A*) and (*g*, *B*) be two bifuzzy soft sets over a common universe *U* with *A*∩*B* ≠ *∅*. Then their *restricted union* is denoted by (*f*, *A*)⋒(*g*, *B*) and is defined as (*f*, *A*)⋒(*g*, *B*) = (*h*, *C*), where *C* = *A*∩*B* and for all *ɛ* ∈ *C*, *h*(*ɛ*) = *f*(*ɛ*) ∪ *g*(*ɛ*).

**Definition 12 (see [7, 14])**

The *extended product* of two bifuzzy soft sets (*f*, *A*) and (*g*, *B*) over *U* is a fuzzy soft set, denoted by (*f*∘*g*, *C*), where *C* = *A* ∪ *B*, and defined by

(7) $$\begin{array}{c} \left(f\circ g\right)\left(ɛ\right)=\{\begin{array}{cc} f\left(ɛ\right) & \text{if}\;ɛ\in A-B,\\ g\left(ɛ\right) & \text{if}\;ɛ\in B-A,\\ f\left(ɛ\right)\circ g\left(ɛ\right) & \text{if}\;ɛ\in A\cap B, \end{array} \end{array}$$

for all *ɛ* ∈ *C*. This is denoted by $\left(f\circ g,C)=(f,A)\;\tilde{\circ }\;(g,B\right)$.

## 3. Bifuzzy Lie Algebras

In this section, we introduce a new kind of generalized bifuzzy Lie algebra, an (*γ*, *δ*)-bifuzzy Lie subalgebra, and discuss some of its properties.

**Definition 13**

A bifuzzy set *A* = (*μ*_*A*_, *ν*_*A*_) in *L* is called an (*γ*, *δ*)-*bifuzzy Lie subalgebra* of *L* if it satisfies the following conditions:

- *x*(*s*_1_, *t*_1_)*γA*,  *y*(*s*_2_, *t*_2_)*γA*⇒(*x* + *y*)(min⁡(*s*_1_, *s*_2_), max⁡(*t*_1_, *t*_2_))*δA*,
- *x*(*s*, *t*)*γA*⇒(*mx*)(*s*, *t*)*δA*,
- *x*(*s*_1_, *t*_1_)*γA*,  *y*(*s*_2_, *t*_2_)*γA*⇒[*x*, *y*](max⁡(*s*_1_, *s*_2_), min⁡(*t*_1_, *t*_2_))*δA*

for all *x*, *y* ∈ *L*,  *m* ∈ *F*,  *s*, *s*_1_, *s*_2_ ∈ (0,1],  *t*, *t*_1_, *t*_2_ ∈ [0,1).

**Remark 14**

Consider (i) *x*(*s*, *t*)*γA*⇒(−*x*)(*s*, *t*)*δA*,

(ii) *x*(*s*, *t*)*γA*⇒(0)(*s*, *t*)*δA*.

**Example 15**

Let *V* be a vector space over a field *F* such that dim⁡(*V*) = 5. Let *V* = {*e*_1_, *e*_2_,…, *e*_5_} be a basis of a vector space over a field *F* with Lie brackets as follows:

(8) [e1,e2]=e3,  [e1,e3]=e5,[e1,e4]=e5,  [e1,e5]=0,[e2,e3]=e5,  [e2,e4]=0,[e2,e5]=0,  [e3,e4]=0,[e3,e5]=0,  [e4,e5]=0,  [ei,ej]=−[ej,ei]

and [*e*_*i*_, *e*_*j*_] = 0 for all *i* = *j*. Then *V* is a Lie algebra over *F*.

We define a bifuzzy set *A* = (*μ*_*A*_, *ν*_*A*_) : *V* → [0,1]×[0,1] by

(9) $$\begin{array}{c} \mu_{A}\left(x\right):=\{\begin{array}{cc} 1 & \text{if}\;x=0,\\ 0.5 & \text{otherwise}, \end{array}\\ \nu_{A}\left(x\right):=\{\begin{array}{cc} 0 & \text{if}\;x=0,\\ 0.3 & \text{otherwise}. \end{array} \end{array}$$

Take *s* = 0.4 ∈ (0,1] and *t* = 0.5 ∈ [1,0). By routine computations, it is easy to see that *A* is not an (*γ*, *δ*)-bifuzzy Lie subalgebra of *L*.

For a bifuzzy set *A* in *L*, we denote *L*(0,1) = {*x* ∈ *L* : *μ*(*x*) > 0 and *ν*(*x*) < 1}.

**Theorem 16**

Let *A* = (*μ*_*A*_, *ν*) be an (*γ*, *δ*)-bifuzzy Lie subalgebra of *L*; then the nonzero set *L*(0,1) is a Lie subalgebra of *L*.

**Proof**

Let *x*, *y* ∈ *L*(0,1). Then *μ*_*A*_(*x*) > 0 and *ν*_*A*_(*x*) < 1, *μ*_*A*_(*y*) > 0 and *ν*_*A*_(*y*) < 1. Assume that *μ*_*A*_(*x* + *y*) = 0 and *ν*_*A*_(*x* + *y*) = 1. If *γ* ∈ {∈, ∈∨*q*}, then we can see that *x*(*μ*_*A*_(*x*), *ν*_*A*_(*x*))*γA* and *y*(*μ*_*A*_(*y*), *ν*_*A*_(*y*))*γA*, but $(x+y)(\min\{\mu_{A}(x),\nu_{A}(x)\},\max\{\mu_{A}(y),\nu_{A}(y)\})\bar{\delta }A$ for all *δ* ∈ {∈, ∈∨*q*, ∈∧*q*}, a contradiction. Also, *x*(1,0)*qA* and *y*(1,0)*qA*, but $(x+y)(1,0)\bar{\delta }A$ for all *δ* ∈ {∈, ∈∨*q*, ∈∧*q*}, a contradiction. Thus *μ*_*A*_(*x* + *y*) > 0 and *ν*_*A*_(*x* + *y*) < 1. Thus *x* + *y* ∈ *L*(0,1). For other conditions the verification is analogous. Consequently *L*(0,1) is a Lie subalgebra of *L*.

**Definition 17**

A bifuzzy set *A* = (*μ*_*A*_, *ν*_*A*_) in *L* is called an (∈, ∈∨*q*)*-bifuzzy Lie algebra* of *L* if it satisfies the following conditions:

(f) *x*(*s*_1_, *t*_1_) ∈ *A*, *y*(*s*_2_, *t*_2_) ∈ *A*⇒(*x* + *y*)(min⁡(*s*_1_, *s*_2_), max⁡(*t*_1_, *t*_2_))∈∨*qA*,
(g) *x*(*s*, *t*) ∈ *A*⇒(*mx*)(*s*, *t*)∈∨*qA*,
(h) *x*(*s*_1_, *t*_1_) ∈ *A*, *y*(*s*_2_, *t*_2_) ∈ *A*⇒[*x*, *y*](max⁡(*s*_1_, *s*_2_), min⁡(*t*_1_, *t*_2_))∈∨*qA*

for all *x*, *y* ∈ *L*, *m* ∈ *F*, *s*, *s*_1_, *s*_2_ ∈ (0,1], *t*, *t*_1_, *t*_2_ ∈ [0,1).

**Theorem 18**

Let *A* = (*μ*_*A*_, *ν*_*A*_) be a bifuzzy set in a Lie algebra *L*. Then *A* is an (∈, ∈∨*q*)-bifuzzy Lie subalgebra of *L* if and only if

- *μ*_*A*_(*x* + *y*)⩾min⁡(*μ*_*A*_(*x*), *μ*_*A*_(*y*), 0.5), *ν*_*A*_(*x* + *y*) ⩽ max⁡(*ν*_*A*_(*x*), *ν*_*A*_(*y*), 0.5),
- *μ*_*a*_(*mx*)⩾min⁡(*μ*_*A*_(*x*), 0.5), *ν*_*a*_(*mx*) ⩽ max⁡(*ν*_*A*_(*x*), 0.5),
- *μ*_*A*_([*x*, *y*])⩾max⁡(*μ*_*A*_(*x*), *μ*_*A*_(*y*), 0.5), *ν*_*A*_([*x*, *y*]) ⩽ min⁡(*ν*_*A*_(*x*), *ν*_*A*_(*y*), 0.5)

hold for all *x*, *y* ∈ *L*, *m* ∈ *F*.

**Proof**

(f)⇒(i): Let *x*, *y* ∈ *L*. We consider the following two cases:

1. min⁡(*μ*_*A*_(*x*), *μ*_*A*_(*y*)) < 0.5, max⁡(*ν*_*A*_(*x*), *ν*_*A*_(*y*)) > 0.5,
2. min⁡(*μ*_*A*_(*x*, *μ*_*A*_(*y*))⩾0.5, max⁡(*ν*_*A*_(*x*, *ν*_*A*_(*y*)) ⩽ 0.5.

*Case *1*.* Assume that *μ*_*A*_(*x* + *y*) < min⁡(*μ*_*A*_(*x*), *μ*_*A*_(*y*), 0.5), *ν*_*A*_(*x* + *y*) > max⁡(*ν*_*A*_(*x*), *ν*_*A*_(*y*), 0.5). Then *μ*_*A*_(*x* + *y*) < min⁡(*μ*_*A*_(*x*), *μ*_*A*_(*y*)), *ν*_*A*_(*x* + *y*) > max⁡(*ν*_*A*_(*x*), *ν*_*A*_(*y*)). Take *s*, *t* such that *μ*_*A*_(*x* + *y*) < *s* < min⁡(*μ*_*A*_(*x*), *μ*_*A*_(*y*)), *ν*_*A*_(*x* + *y*) > *t* > max⁡(*ν*_*A*_(*x*), *ν*_*A*_(*y*)). Then *x*_*s*_, *y*_*s*_ ∈ *μ*_*A*_ and *x*_*t*_, *y*_*t*_ ∈ *ν*_*A*_, but $\left(x+y\right)\left(\min\left(s_{1},s_{2}\right),\max\left(t_{1},t_{2}\right)\right)\bar{\in \vee q}A$, which is contradiction with (*f*).

*Case *2*.* Assume that *μ*_*A*_(*x* + *y*) < 0.5, *ν*_*A*_(*x* + *y*) > 0.5. Then *x*(0.5,0.5), *y*(0.5,0.5) ∈ *A* but $(x+y)(0.5,0.5)\bar{\in \vee q}A$, a contradiction. Hence (i) holds. (i)⇒(f): Let *x*(*s*_1_, *t*_1_), *y*(*s*_2_, *t*_2_) ∈ *A*; then *μ*_*A*_(*x*)⩾*s*_1_, *μ*_*A*_(*y*)⩾*s*_2_, *ν*_*A*_(*x*) ⩽ *t*_1_, *ν*_*A*_(*y*) ⩽ *t*_2_. Now, we have

(10) $$\begin{array}{c} \mu_{A}\left(x+y\right)⩾\min\left(\mu_{A}\left(x\right),\mu_{A}\left(y\right),0.5\right)⩾\min\left(s_{1},s_{2},0.5\right),\\ \nu_{A}\left(x+y\right)⩽\max\left(\nu_{A}\left(x\right),\nu_{A}\left(y\right),0.5\right)⩽\max\left(t_{1},t_{2},0.5\right). \end{array}$$

If min⁡(*s*_1_, *s*_2_) > 0.5, max⁡(*t*_1_, *t*_2_ < 0.5, then *μ*_*A*_(*x* + *y*)⩾0.5⇒*μ*_*A*_(*x* + *y*) + min⁡(*s*_1_, *s*_2_) > 1, *ν*_*A*_(*x* + *y*) ⩽ 0.5⇒*ν*_*A*_(*x* + *y*) + max⁡(*t*_1_, *t*_2_) < 1. On the other hand, if min⁡(*s*_1_, *s*_2_) ⩽ 0.5, max⁡(*t*_1_, *t*_2_)⩾0.5, then *μ*_*A*_(*x* + *y*)⩾min⁡(*s*_1_, *s*_2_), *ν*_*A*_(*x* + *y*) ⩽ max⁡(*t*_1_, *t*_2_). Hence (*x* + *y*)(min⁡(*s*_1_, *s*_2_), max⁡(*t*_1_, *t*_2_))∈∨*qA*. The verification of (g)⇔(j) and (h)⇔(k) is analogous and we omit the details. This completes the proof.

**Theorem 19**

Let *A* = (*μ*_*A*_, *ν*_*A*_) be a bifuzzy set of Lie algebra of *L*. Then *A* is an (∈, ∈∨*q*)-bifuzzy Li subalgebra of *L* if and only if each nonempty *A*_(*s*,*t*)_, *s* ∈ (0.5,1], *t* ∈ [0.5,1) is a Lie subalgebra of *L*.

**Proof**

Assume that *A* = (*μ*_*A*_, *ν*_*A*_) is an (∈, ∈∨*q*)-bifuzzy Lie subalgebra of *L* and let *s* ∈ (0.5,1], *t* ∈ [0.5,1). If *x*, *y* ∈ *A*_(*s*,*t*)_ and *m* ∈ *F*, then *μ*_*A*_(*x*) ≥ *s* and *μ*_*A*_(*y*) ≥ *s*, *ν*_*A*_(*x*) ≤ *t* and *ν*_*A*_(*y*) ≤ *t*. Thus,

(11) $$\begin{array}{c} \mu_{A}\left(x+y\right)⩾\min\left(\mu_{A}\left(x\right),\mu_{A}\left(y\right),0.5\right)⩾\min\left(s,0.5\right)=s,\\ \nu_{A}\left(x+y\right)⩽\max\left(\nu_{A}\left(x\right),\nu_{A}\left(y\right),0.5\right)⩽\max\left(t,0.5\right)=t,\\ \mu_{A}\left(mx\right)⩾\min\left(\mu_{A}\left(x\right),0.5\right)⩾\min\left(s,0.5\right)=s,\\ \nu_{A}\left(mx\right)⩽\max\left(\nu_{A}\left(x\right),0.5\right)⩽\max\left(t,0.5\right)=t,\\ \mu_{A}\left(\left[x,y\right]\right)⩾\min\left(\mu_{A}\left(x\right),\mu_{A}\left(y\right),0.5\right)⩾\min\left(t,0.5\right)=t,\\ \nu_{A}\left(\left[x,y\right]\right)⩽\max\left(\nu_{A}\left(x\right),\nu_{A}\left(y\right),0.5\right)⩽\max\left(t,0.5\right)=t, \end{array}$$

and so *x* + *y*, *mx*, [*x*, *y*] ∈ *A*_(*s*,*t*)_. This shows that *L*(*ν*; *t*) are Lie subalgebras of *L*. The proof of converse part is obvious. This ends the proof.

**Theorem 20**

Let *A* be a bifuzzy set in a Lie algebra *L*. Then *A*_(*s*,*t*)_ is a Lie subalgebra of *L* if and only if

1. max⁡(*μ*_*A*_(*x* + *y*), 0.5)⩾min⁡(*μ*_*A*_(*x*), *μ*_*A*_(*y*)), min⁡(*ν*_*A*_(*x* + *y*), 0.5) ⩽ max⁡(*ν*_*A*_(*x*), *ν*_*A*_(*y*)),
2. max⁡(*μ*_*A*_(*mx*), 0.5)⩾*μ*_*A*_(*x*), min⁡(*ν*_*A*_(*mx*), 0.5) ⩽ *ν*_*A*_(*x*),
3. max⁡(*μ*_*A*_([*x*, *y*]), 0.5))⩾min⁡(*μ*_*A*_(*x*), *μ*_*A*_(*y*)), min⁡(*ν*_*A*_([*x*, *y*]), 0.5)) ⩽ max⁡(*ν*_*A*_(*x*), *ν*_*A*_(*y*)) for all *x*, *y* ∈ *L*, *m* ∈ *F*.

**Proof**

Suppose that *A*_(*s*,*t*)_ is a Lie subalgebra of *L*. Let max⁡(*μ*_*A*_(*x* + *y*), 0.5) < min⁡(*μ*_*A*_(*x*), *μ*_*A*_(*y*)) = *s*, min⁡(*ν*_*A*_(*x* + *y*), 0.5) > max⁡(*ν*_*A*_(*x*), *ν*_*A*_(*y*)) = *t* for some *x*, *y* ∈ *L*; then *s* ∈ (0.5,1], *t* ∈ [0.5,1), *μ*_*A*_(*x* + *y*) < *s*, *ν*_*A*_(*x* + *y*) > *t*, *x*, *y* ∈ *A*_(*s*,*t*)_. Since *x*, *y* ∈ *A*_(*s*,*t*)_ and *A*_(*s*,*t*)_ is a Lie subalgebra of *L*, so *x* + *y* ∈ *A*_(*s*,*t*)_ or *μ*_*A*_(*x* + *y*)⩾*s*, *ν*_*A*_(*x* + *y*) ⩽ *t*, which is contradiction with *μ*_*A*_(*x* + *y*) < *s*, *ν*_*A*_(*x* + *y*) > *t*. Hence (1) holds. For (2), (3) the verification is analogous.

Conversely, suppose that (1)–(3) hold. Assume that *s* ∈ (0.5,1], *t* ∈ [0.5,1), *x*, *y* ∈ *A*_(*s*,*t*)_. Then

(12) 0.5<s⩽min⁡(μA(x),μA(y))⩽max⁡(μA(x+y),0.5)⇒μA(x+y)⩾s,0.5>t⩾max⁡(νA(x),νA(y))⩾min⁡(νA(x+y),0.5)⇒νA(x+y)⩽t,0.5<s⩽μA(x)⩽max⁡(μA(mx),0.5)⇒μA(mx)⩾s,0.5>t⩾νA(x)⩾min⁡(νA(mx),0.5)⇒νA(mx)⩽t,0.5<s⩽min⁡(μA(x),μA(y))⩽max⁡(μA[x,y],0.5)⇒μA([x,y])⩾s,0.5>t⩾max⁡(νA(x),νA(y))⩾min⁡(νA[x,y],0.5)⇒νA([x,y])⩽t,

and so *x* + *y*, *mx*, [*x*, *y*] ∈ *A*_(*s*,*t*)_. This shows that *A*_(*s*,*t*)_ is a Lie subalgebra of *L*.

**Theorem 21**

The intersection of any family of (∈, ∈∨*q*)-bifuzzy Lie subalgebras of *L* is an (∈, ∈∨*q*)-bifuzzy Lie subalgebra.

**Proof**

Let {*A*_*i*_ : *i* ∈ Λ} be a family of (∈, ∈∨*q*)-bifuzzy Lie subalgebra of *L* and let *A* : = ⋂_*i*∈Λ_ *A*_*i*_ = (sup⁡_*i*∈Λ_ *μ*_*i*_, inf⁡_*i*∈Λ_ *ν*_*i*_). Let *x*, *y* ∈ *L*, then by Theorem 19, we have *μ*_*A*_(*x* + *y*)⩾min⁡(*μ*_*A*_(*x*), *μ*_*A*_(*y*), 0.5), *ν*_*A*_(*x* + *y*) ⩽ max⁡(*ν*_*A*_(*x*), *ν*_*A*_(*y*), 0.5), and hence

(13) μA(x+y)=sup⁡i∈Λ⁡ μi(x+y)⩾sup⁡i∈Λ⁡min⁡(μi(x),μi(y),0.5)=min⁡(sup⁡i∈Λ⁡μi(x),sup⁡i∈Λ⁡ μi(y),0.5)=min⁡(⋂i∈Λμi(x),⋂i∈Λμi(y),0.5)=min⁡(μA(x),μA(y),0.5),νA(x+y)=inf⁡i∈Λ νi(x+y)⩽inf⁡i∈Λ⁡max⁡(νi(x),νi(y),0.5)=max⁡(inf⁡i∈Λ⁡ νi(x),inf⁡i∈Λ⁡ νi(y),0.5)=max⁡(⋂i∈Λνi(x),⋂i∈Λνi(y),0.5)=max⁡(νA(x),νA(y),0.5).

For other conditions the verification is analogous. By Theorem 19, it follows that *A* is an (∈, ∈∨*q*)-bifuzzy Lie subalgebra of *L*.

**Theorem 22**

Let *L*_0_ ⊂ *L*_1_ ⊂ ⋯⊂*L*_*n*_ = *L* be a strictly increasing chain of (∈, ∈)-bifuzzy Lie subalgebras of a Lie algebra *L*; then there exists (∈, ∈)-bifuzzy Lie subalgebra *A* = (*μ*_*A*_, *ν*_*A*_) of *L* whose level subalgebras are precisely the members of the chain with *A*_0.5_ = (*μ*_0.5_, *ν*_0.5_) = *L*(0,1).

For any *r*, *s* ∈ [0,1] and fuzzy subset *μ* in *L*, denotes $\mu_{\hat{r}}=\{x\in L|\mu (x)>r\}$, 〈*μ*〉_*r*_ = {*x* ∈ *L* | *x*_*r*_ *qμ*}, [*μ*]_*r*_ = {*x* ∈ *L* | *x*_*r*_ ∈ ∨*qμ*}, ${\hat{\nu }}_{s}=\{x\in L|\mu (x)<s\}$, and ${\hat{\left[\nu \right]}}_{s}=\{x\in L|x_{s}\bar{\in }\vee \bar{q}\nu \}$. Clearly, $A^{\left(r,s\right)}=\mu_{A\hat{r}}\cap {\hat{\mu_{A}}}_{s}$ for all *r*, *s* ∈ [0,1].

We state here a nice characterization without proof.

**Theorem 23**

Let *L* be a Lie algebra and *A* a bifuzzy set in *L*. Then

- *A* is an (∈, ∈∨*q*)-bifuzzy Lie subalgebra of *L* if and only if nonempty subsets $\mu_{A\hat{r}}$ and ${\hat{\nu_{A}}}_{s}$ are Lie subalgebras of *L* for all *r* ∈ [0,0.5) and *s* ∈ (0.5,1];
- *A* is an (∈, ∈∨*q*)-bifuzzy Lie subalgebra of *L* if and only if nonempty subsets $\mu_{A\hat{r}}$ and ${\hat{\left[\nu_{A}\right]}}_{s}$ are Lie subalgebras of *L* for all *r* ∈ [0,0.5) and *s* ∈ (0,1];
- *A* is an (∈, ∈∨*q*)-intuitionistic fuzzy Lie subalgebra of *L* if and only if nonempty subsets 〈*μ*_*A*_〉_*r*_ and ${\hat{\nu_{A}}}_{s}$ are *K*-subalgebras of *𝒦* for all *r*, *s* ∈ (0.5,1];
- *A* is an (∈, ∈∨*q*)-bifuzzy Lie subalgebra of *L* if and only if nonempty subsets 〈*μ*_*A*_〉_*r*_ and ${\hat{\left[\nu_{A}\right]}}_{s}$ are Lie subalgebras of *L* for all *r* ∈ (0.5,1] and *s* ∈ (0,1];
- *A* is an (∈, ∈∨*q*)-bifuzzy Lie subalgebra of *L* if and only if nonempty subsets [*μ*_*A*_]_*r*_ and ${\hat{\nu_{A}}}_{s}$ are Lie subalgebras of *L* for all *r* ∈ (0,1] and *s* ∈ (0.5,1];
- *A* is an (∈, ∈∨*q*)-bifuzzy Lie subalgebra of *L* if and only if nonempty subsets [*μ*_*A*_]_*r*_ and ${\left[\hat{\nu_{A}}\right]}_{s}$ are ideals of *L* for all *r*, *s* ∈ (0,1].

## 4. Bifuzzy Soft Lie Algebras

In this section, we introduce bifuzzy soft Lie subalgebras and investigate some of their properties.

**Definition 24**

Let *L* be a Lie algebra and let (*f*, *A*) be a bifuzzy soft set over *L*. Then (*f*, *A*) is said to be a *bifuzzy soft Lie subalgebra* over *L* if *f*(*x*) is a bifuzzy Lie subalgebra of *L* for all *x* ∈ *A*; that is, a bifuzzy soft set (*f*, *A*) on *L* is called a *bifuzzy soft Lie subalgebra* of *L* if

- *μ*_*f*_*ɛ*__(*x* + *y*)⩾min⁡{*μ*_*f*_*ɛ*__(*x*), *μ*_*f*_*ɛ*__(*y*)},
- *ν*_*f*_*ɛ*__(*x* + *y*) ⩽ max⁡{*ν*_*f*_*ɛ*__(*x*), *ν*_*f*_*ɛ*__(*y*)},
- *μ*_*f*_*ɛ*__(*mx*)⩾*μ*_*f*_*ɛ*__(*x*),
- *ν*_*f*_*ɛ*__(*mx*) ⩽ *μ*_*f*_*ɛ*__(*x*),
- *μ*_*f*_*ɛ*__([*x*, *y*])⩾min⁡{*μf*_*ɛ*_(*x*), *μ*_*f*_*ɛ*__(*y*)},
- *ν*_*f*_*ɛ*__([*x*, *y*]) ⩽ max⁡{*νf*_*ɛ*_(*x*), *ν*_*f*_*ɛ*__(*y*)}

hold for all *x*, *y* ∈ *L* and *m* ∈ *K*.

**Example 25**

Let *ℜ* ^2^ = {(*x*, *y*) : *x*, *y* ∈ ℝ} be the set of all 2-dimensional real vectors. Then *ℜ* ^2^ with [*x*, *y*] = *x* × *y* is a real Lie algebra. Let *ℕ* and *ℤ* denote the set of all natural numbers and the set of all integers, respectively. Define *f* : *ℤ* → ([0,1]×[0,1])^*ℜ*^2^^ by *f*(*n*) = *f*_*n*_ : *ℜ* ^2^ → [0,1] × [0,1] for all *n* ∈ *ℤ*,

(14) $$\begin{array}{c} \mu_{f_{n}}\left(x\right)=\{\begin{array}{cc} 0.6 & \text{if}\;x=\left(0,0\right)=\underline{0},\\ 0.2 & \text{if}\;x=\left(0,a\right),\;a\neq 0,\\ 0 & \text{otherwise}, \end{array}\\ \nu_{f_{n}}\left(x\right)=\{\begin{array}{cc} 0.0 & \text{if}\;x=\left(0,0\right)=\underline{0},\\ 0.4 & \text{if}\;x=\left(0,a\right),a\neq 0,\\ 0.6 & \text{otherwise}. \end{array} \end{array}$$

By routine computations, we can easily check that (*f*, *ℤ*) is a bifuzzy soft Lie subalgebra of *ℜ* ^2^.

The following proposition is obvious.

**Proposition 26**

Let (*f*, *A*) be a bifuzzy soft Lie subalgebra of *L*; then

- $\mu_{f_{ɛ}}\left(\underline{0}\right)⩾\mu_{f_{ɛ}}\left(x\right)$,
- $\nu_{f_{ɛ}}(\underline{0})⩽\nu_{f_{ɛ}}(x)$

for all *x* ∈ *L*.

**Definition 27**

Let (*f*, *A*) be a bifuzzy soft set over *U*. For each *s*, *t* ∈ [0,1], the set (*f*, *A*)^(*s*,*t*)^ = (*f* ^(*s*,*t*)^, *A*) is called an (*s*, *t*)-level soft set of (*f*, *A*), where *f*_*ɛ*_^(*s*,*t*)^ = {*x* ∈ *U* | *μ*_*f*_*ɛ*__(*x*) ≥ *s*, *ν*_*f*_*ɛ*__(*x*) ≤ *t*} for all *ɛ* ∈ *A*.

**Theorem 28**

Let (*f*, *A*) be a bifuzzy soft set over *L*. (*f*, *A*) is a bifuzzy soft Lie subalgebra if and only if (*f*, *A*)^(*s*,*t*)^ is a soft Lie subalgebra over *L* for each *s*, *t* ∈ [0,1].

**Proof**

Suppose that (*f*, *A*) is a bifuzzy soft Lie subalgebra. For each *s*, *t* ∈ [0,1], *ɛ* ∈ *A*, and *x*_1_, *x*_2_ ∈ (*f*, *A*)_*ɛ*_^(*s*,*t*)^, then *μ*_*f*_*ɛ*__(*x*_1_) ≥ *s*, *μ*_*f*_*ɛ*_(*x*_2_)_ ≥ *s* and *ν*_*f*_*ɛ*__(*x*_1_) ≤ *t*, *ν*_*f*_*ɛ*_(*x*_2_)_ ≤ *t*. From Definition 27, it follows that (*f*, *A*)_*ɛ*_^(*s*,*t*)^ is a bifuzzy Lie subalgebra over *L*. Thus *μ*_*f*_*ɛ*__(*x*_1_ + *x*_2_) ≥ min⁡(*μ*_*f*_*ɛ*__(*x*_1_), *μ*_*f*_*ɛ*__(*x*_2_)), *μ*_*f*_*ɛ*__(*x*_1_ + *x*_2_) ≥ *s*, *ν*_*f*_*ɛ*__(*x*_1_ + *x*_2_) ≤ max⁡(*ν*_*f*_*ɛ*__(*x*_1_), *ν*_*f*_*ɛ*__(*x*_2_)), *ν*_*f*_*ɛ*__(*x*_1_ + *x*_2_) ≤ *t*. This implies that *x*_1_ + *x*_2_ ∈ (*f*, *A*)_*ɛ*_^(*s*,*t*)^. Verification for other conditions is similar. Hence we omit the details. This shows that (*f*, *A*)_*ɛ*_^(*s*,*t*)^ is a Lie subalgebra over *L*. According to Definition 27, (*f*, *A*)^(*s*,*t*)^ is a soft Lie subalgebra over *L* for each *s*, *t* ∈ [0,1].

Conversely, assume that (*f*, *A*)^(*s*,*t*)^ is a soft Lie subalgebra over *L* for each *s*, *t* ∈ [0,1]. For each *ɛ* ∈ *A* and *x*_1_, *x*_2_ ∈ *L*, let *s* = min⁡{*μ*_*f*_*ɛ*__(*x*_1_), *μ*_*f*_*ɛ*__(*x*_2_)} and let *t* = max⁡{*ν*_*f*_*ɛ*__(*x*_1_), *ν*_*f*_*ɛ*__(*x*_2_)}; then *x*_1_, *x*_2_ ∈ (*f*, *A*)_*ɛ*_^(*s*,*t*)^. Since (*f*, *A*)_*ɛ*_^(*s*,*t*)^ is a Lie subalgebra over *L*, then *x*_1_ + *x*_2_ ∈ (*f*, *A*)_*ɛ*_^(*s*,*t*)^. This means that *μ*_*f*_*ɛ*__(*x*_1_ + *x*_2_) ≥ min⁡(*μ*_*f*_*ɛ*__(*x*_1_), *μ*_*f*_*ɛ*__(*x*_2_)), *ν*_*f*_*ɛ*__(*x*_1_ + *x*_2_) ≤ max⁡(*ν*_*f*_*ɛ*__(*x*_1_), *ν*_*f*_*ɛ*__(*x*_2_)). Verification for other conditions is similar. Hence we omit the details. Thus, (*f*, *A*)_*ɛ*_^(*s*,*t*)^ is a bifuzzy Lie subalgebra over *L*. According to Definition 24, (*f*, *A*) is a bifuzzy soft Lie subalgebra over *L*. This completes the proof.

**Definition 29**

Let *ϕ* : *X* → *Y* and *ψ* : *A* → *B* be two functions, where *A* and *B* are parametric sets from the crisp sets *X* and *Y*, respectively. Then the pair (*ϕ*, *ψ*) is called a *bifuzzy soft function* from *X* to *Y*.

**Definition 30**

Let (*f*, *A*) and (*g*, *B*) be two bifuzzy soft sets over *L*_1_ and *L*_2_, respectively, and let (*ϕ*, *ψ*) be a bifuzzy soft function from *L*_1_ to *L*_2_.

(1) The image of (*f*, *A*) under the bifuzzy soft function (*ϕ*, *ψ*), denoted by (*ϕ*, *ψ*)(*f*, *A*), is the bifuzzy soft set on *L*_2_ defined by (*ϕ*, *ψ*)(*f*, *A*) = (*ϕ*(*f*), *ψ*(*A*)), where for all *k* ∈ *ψ*(*A*), *y* ∈ *L*_2_
  (15) $$\begin{array}{c} \mu_{\phi {\left(f\right)}_{k}}\left(y\right)=\{\begin{array}{cc} \overset{}{\underset{\phi \left(x\right)=y}{⋁}}\overset{}{\underset{\psi \left(a\right)=k}{⋁}}f_{a}\left(x\right) & \text{if}\;x\in \psi^{-1}\left(y\right),\\ 1 & \text{otherwise}, \end{array}\\ \nu_{\phi {\left(f\right)}_{k}}\left(y\right)=\{\begin{array}{cc} \overset{}{\underset{\phi \left(x\right)=y}{⋀}}\overset{}{\underset{\psi \left(a\right)=k}{⋀}}f_{a}\left(x\right) & \text{if}\;x\in \psi^{-1}\left(y\right),\\ 0 & \text{otherwise}. \end{array} \end{array}$$
(2) The preimage of (*g*, *B*) under the bifuzzy soft function (*ϕ*, *ψ*), denoted by (*ϕ*, *ψ*)^−1^(*g*, *B*), is the bifuzzy soft set over *L*_1_ defined by (*ϕ*, *ψ*)^−1^(*g*, *B*) = (*ϕ* ^−1^(*g*), *ψ* ^−1^(*B*)), where for all *a* ∈ *ψ* ^−1^(*A*), for all *x* ∈ *L*_1_,
  (16) $$\begin{array}{c} \mu_{\phi^{-1}{\left(g\right)}_{a}}\left(x\right)=\mu_{g_{\psi (a)}}\left(\phi \left(x\right)\right),\\ \nu_{\phi^{-1}{\left(g\right)}_{a}}\left(x\right)=\nu_{g_{\psi (a)}}\left(\phi \left(x\right)\right). \end{array}$$

**Definition 31**

Let (*ϕ*, *ψ*) be a bifuzzy soft function from *L*_1_ to *L*_2_. If *ϕ* is a homomorphism from *L*_1_ to *L*_2_, then (*ϕ*, *ψ*) is said to be *bifuzzy soft homomorphism*. If *ϕ* is a isomorphism from *L*_1_ to *L*_2_ and *ψ* is one-to-one mapping from *A* onto *B* then (*ϕ*, *ψ*) is said to be bifuzzy soft isomorphism.

**Theorem 32**

Let (*g*, *B*) be a bifuzzy soft Lie subalgebra over *L*_2_ and let (*ϕ*, *ψ*) be a bifuzzy soft homomorphism from *L*_1_ to *L*_2_. Then (*ϕ*, *ψ*)^−1^(*g*, *B*) is a bifuzzy soft Lie subalgebra over *L*_1_.

**Proof**

Let *x*_1_, *x*_2_ ∈ *L*_1_; then

(17) ϕ−1(μgɛ)(x1+x2)  =μgψ(ɛ)(ϕ(x1+x2))=μgψ(ɛ)(ϕ(x1)+ϕ(x2))  ⩾min⁡{μgψ(ɛ)(ϕ(x1)),μgψ(ɛ)(ϕ(x2))}  =min⁡{ϕ−1(μgɛ)(x1),ϕ−1(μgɛ)(x2)},ϕ−1(νgɛ)(x1+x2)  =νgψ(ɛ)(ϕ(x1+x2))=νgψ(ɛ)(ϕ(x1)+ϕ(x2))  ⩽max⁡{νgψ(ɛ)(ϕ(x1)),νgψ(ɛ)(ϕ(x2))}  =max⁡{ϕ−1(νgɛ)(x1),ϕ−1(νgɛ)(x2)}.

Verification for other conditions is similar. Hence we omit the details. Hence (*ϕ*, *ψ*)^−1^(*g*, *B*) is a bifuzzy soft Lie subalgebra over *L*_1_.

**Remark 33**

Let (*f*, *A*) be a bifuzzy soft Lie subalgebra over *L*_1_ and let (*ϕ*, *ψ*) be a bifuzzy soft homomorphism from *L*_1_ to *L*_2_. Then (*ϕ*, *ψ*)(*f*, *A*) may not be a bifuzzy soft Lie subalgebra over *L*_2_.

**Theorem 34**

Let (*f*, *A*) be a bifuzzy soft Lie subalgebra over *L* and let {(*h*_*i*_, *B*_*i*_) | *i* ∈ *I*} be a nonempty family of bifuzzy soft Lie subalgebras of (*f*, *A*). Then

- ${\tilde{⋂}}_{i\in I}(h_{i},B_{i})$ is a bifuzzy soft Lie subalgebra of (*f*, *A*);
- ⋀_*i*∈*I*_(*h*_*i*_, *B*_*i*_) is a bifuzzy soft Lie subalgebra of ⋀_*i*∈*I*_(*f*, *A*);
- if *B*_*i*_∩*B*_*j*_ = *∅* for all *i*, *j* ∈ *I*, then ${\tilde{⋁}}_{i\in I}(H_{i},B_{i})$ is an bifuzzy soft Lie subalgebra of ${\tilde{⋁}}_{i\in I}(f,A)$.

As a generalization of above theorem, we have the following result.

**Theorem 35**

Let (*f*, *A*) be an (∈, ∈∨*q*)-bifuzzy soft Lie subalgebra over *L* and let {(*h*_*i*_, *B*_*i*_) | *i* ∈ *I*} be a nonempty family of (∈, ∈∨*q*)-bifuzzy soft *K*-subalgebras of (*f*, *A*); then

- ${\tilde{⋂}}_{i\in I}(h_{i},B_{i})$ is an (∈, ∈∨*q*)-bifuzzy soft Lie subalgebra of (*f*, *A*);
- ⋀_*i*∈*I*_(*h*_*i*_, *B*_*i*_) is an (∈, ∈∨*q*)-bifuzzy soft Lie subalgebra of ⋀_*i*∈*I*_(*f*, *A*);
- if *B*_*i*_∩*B*_*j*_ = *∅* for all *i*, *j* ∈ *I*, then ${\tilde{⋁}}_{i\in I}(H_{i},B_{i})$ is an (∈, ∈∨*q*)- bifuzzy soft Lie subalgebra of ${\tilde{⋁}}_{i\in I}(f,A)$.

**Theorem 36**

Let (*f*, *A*) and (*g*, *B*) be two (∈, ∈∨*q*)-bifuzzy soft *K*-subalgebras over a Lie algebra *L*. Then $\left(f,A)\;\tilde{\cap }\;(g,B\right)$ is an (∈, ∈∨*q*)-bifuzzy soft Lie subalgebra over *L*.

**Proof**

By Definition 12, we can write $\left(f,A)\;\tilde{\cap }\;(g,B)=(h,C\right)$, where *C* = *A* ∪ *B* and

(18) $$\begin{array}{c} h\left(\alpha \right)=\{\begin{array}{cc} f\left(\alpha \right) & \text{if}\;\alpha \in A-B,\\ g\left(\alpha \right) & \text{if}\;\alpha \in B-C,\\ f\left(\alpha \right)\cap g\left(\alpha \right) & \text{if}\;\alpha \in A\cap B, \end{array} \end{array}$$

for all *α* ∈ *C*.

Now for any *α* ∈ *C*, we consider the following cases.

*Case *1*.* Consider (*α* ∈ *A* − *B*). Then *h*(*α*) = *f*(*α*) is an (∈, ∈∨*q*)-bifuzzy Lie subalgebra of *L* since (*f*, *A*) is an (∈, ∈∨*q*)-bifuzzy soft Lie subalgebra over *L*.

*Case *2*.* Consider (*α* ∈ *B* − *A*). Then *h*(*α*) = *g*(*α*) is an (∈, ∈∨*q*)-bifuzzy Lie subalgebra of *L* since (*g*, *B*) is an (∈, ∈∨*q*)-bifuzzy soft Lie subalgebra over *L*.

*Case *3*.* Consider (*α* ∈ *A*∩*B*). Then *h*(*α*) = *f*(*α*)∩*g*(*α*) is an (∈, ∈∨*q*)-bifuzzy Lie subalgebra of *L* by the assumption. Thus, in any case, *h*(*α*) is an (∈, ∈∨*q*)-bifuzzy Lie subalgebra of *L*. Therefore, $\left(f,A)\;\tilde{\cap }\;(g,B\right)$ is an (∈, ∈∨*q*)-bifuzzy soft Lie subalgebra over *L*.

**Theorem 37**

Let (*f*, *A*) and (*g*, *B*) be two (∈, ∈∨*q*)-bifuzzy soft *K*-subalgebras over a Lie algebra *L*. If *A* and *B* are disjoint, then $\left(f,A)\;\tilde{\cup }\;(g,B\right)$ is an (∈, ∈∨*q*)-bifuzzy softLie subalgebra over *L*.

**Lemma 38**

Let *L* be a Lie algebra and (*f*, *A*) and (*g*, *B*) intuitionistic bifuzzy soft sets on *G*. If (*f*, *A*) and (*g*, *B*) are (∈, ∈∨*q*)-intuitionistic bifuzzy soft Lie subalgebra on *L*, then so are (*f*, *A*)∩(*g*, *B*) and $\left(f,A)\;\tilde{\cap }\;(g,B\right)$. Moreover, if (*f*, *A*) and (*g*, *B*) are an (∈, ∈∨*q*)-bifuzzy soft Lie subalgebra on *L* and an (∈, ∈∨*q*)-bifuzzy soft Lie subalgebra on *L*, then $\left(f,A){⊙}_{h}(g,B)\subset (f,A)\;\tilde{\cap }\;(g,B\right)$.

**Lemma 39**

Let *L* be a Lie algebra and (*f*, *A*) and (*g*, *B*) bifuzzy soft sets on *L*. If (*f*, *A*) and (*g*, *B*) are (∈, ∈∨*q*)-bifuzzy soft Lie subalgebra on *L*, then so are (*f*, *A*)∪(*g*, *B*) and $\left(f,A)\;\tilde{\cup }\;(g,B\right)$.

Denote by *𝕊𝕀*(*G*, *E*) the set of all (∈, ∈∨*q*)-bifuzzy soft Lie subalgebras on *L*.

**Theorem 40**

$\left(𝔽𝕊𝕀(G,E),\tilde{\cup },\cap \right)$ is a complete distributive lattice under the ordering relation ⊂.

**Proof**

For any (*f*, *A*), (*g*, *A*) ∈ *𝔽* *𝕊𝕀*(*G*, *E*), by above Lemmas, $\left(f,A)\;\tilde{\cup }\;(g,A)\in 𝔽𝕊𝕀(G,E\right)$ and (*f*, *A*)∩(*g*, *A*) ∈ *𝔽* *𝕊𝕀*(*G*, *E*). It is obvious that $\left(f,A)\;\tilde{\cup }\;(g,A\right)$ and (*f*, *A*)∩(*g*, *A*) are the least upper bound and the greatest lower bound of (*f*, *A*) and (*g*, *B*), respectively. There is no difficulty in replacing {(*f*, *A*), (*g*, *A*)} with an arbitrary family of *𝔽* *𝕊𝕀*(*G*, *E*) and so $\left(𝔽𝕊𝕀(G,E),\tilde{\cup },\cap \right)$ is a complete lattice. Now we prove that the following distributive law

(19) (f,A)∩((g,A) ∪~ (h,C))  =((f,A)∩(g,A)) ∪~ ((f,A)∩(h,C))

holds for all (*f*, *A*), (*g*, *A*), (*h*, *C*) ∈ *𝔽* *𝕊𝕀*(*G*, *E*). Suppose that

(20) (f,A)∩((g,A) ∪~ (h,C))=(I,A∩(B∪C)),((f,A)∩(g,B)) ∪~ ((f,A)∩(h,C))=(J,(A∩B)∪(A∩C))=(J,A∩(B∪C)).

Now for any *ɛ* ∈ *A*∩(*B* ∪ *C*), it follows that *ɛ* ∈ *A* and *ɛ* ∈ *B* ∪ *C*. We consider the following cases.

*Case *1*.* Consider (*ɛ* ∈ *A*, *ɛ* ∉ *B* and *ɛ* ∈ *C*). Then *I*(*ɛ*) = *f*(*ɛ*)∩*h*(*ɛ*) = *J*(*ɛ*).

*Case *2*.* Consider (*ɛ* ∈ *A*, *ɛ* ∈ *B* and *ɛ* ∉ *C*). Then *I*(*ɛ*) = *f*(*ɛ*)∩*G*(*ɛ*) = *J*(*ɛ*).

*Case *3*.* Consider (*ɛ* ∈ *A*, *ɛ* ∈ *B* and *ɛ* ∈ *C*). Then *I*(*ɛ*) = *f*(*ɛ*)∩(*g*(*ɛ*) ∪ *h*(*ɛ*)) = (*f*(*ɛ*)∩*g*(*ɛ*)) ∪ (*f*(*ɛ*)∩*h*(*ɛ*)) = *J*(*ɛ*).

Therefore, *I* and *J* are the same operators, and so $(f,A)\cap ((g,A)\;\tilde{\cup }\;(h,C))\;=$ $\left((f,A)\cap (g,B))\;\tilde{\cup }\;((f,A)\cap (h,C)\right)$. It follows that $\left(f,A\right)\cap \left(\left(g,A\right)\;\tilde{\cup }\;\left(h,C\right)\right)\;=$ $\left((f,A)\cap (g,B))\;\tilde{\cup }\;((f,A)\cap (h,C)\right)$. This completes the proof.

**Definition 41**

The product of two bifuzzy soft sets (*f*, *A*) and (*g*, *A*) over a Lie algebra is a bifuzzy soft set over *G*, denoted by (*f*∘*g*, *C*), where *C* = *A* ∪ *B* and

(21) $$\begin{array}{c} \left(f\circ g\right)\left(ɛ\right)=\{\begin{array}{cc} f\left(ɛ\right) & \text{if}\;ɛ\in A-B,\\ g\left(ɛ\right) & \text{if}\;ɛ\in B-A,\\ f\left(ɛ\right)\circ G\left(ɛ\right) & \text{if}\;ɛ\in A\cap B, \end{array} \end{array}$$

for all *ɛ* ∈ *C*. This is denoted by (*f*∘*g*, *C*) = (*f*, *A*)⊙(*g*, *A*).

The following results can be easily deduced.

**Lemma 42**

Let (*f*_1_, *A*), (*f*_2_, *A*), (*g*_1_, *B*), and (*g*_2_, *B*) be bifuzzy soft sets over a Lie algebra *L* such that (*f*_1_, *A*)⊂(*f*_2_, *A*) and (*g*_1_, *B*)⊂(*g*_2_, *B*). Then

- (*f*_1_, *A*)⊙(*g*_1_, *B*)⊂(*f*_2_, *A*)⊙(*g*_2_, *B*),
- (*f*_1_, *A*)∩(*g*_1_, *B*)⊂(*f*_2_, *A*)∩(*g*_2_, *B*) and $\left(f_{1},A)\;\tilde{\cap }\;(g_{1},B)\subset (f_{2},A)\;\tilde{\cap }\;(g_{2},B\right)$,
- (*f*_1_, *A*)∪(*g*_1_, *B*)⊂(*f*_2_, *A*)∪(*g*_2_, *B*) and $\left(f_{1},A)\;\tilde{\cup }\;(g_{1},B)\subset (f_{2},A)\;\tilde{\cup }\;(g_{2},B\right)$.

**Lemma 43**

Let (*f*, *A*), (*g*, *A*), and (*h*, *C*) be bifuzzy soft sets over a Lie algebra *L*. Then (*F*, *A*)⊙((*g*, *A*)⊙(*h*, *C*)) = ((*f*, *A*)⊙(*g*, *B*))⊙(*h*, *C*).

Now we consider the bifuzzy soft sets over a definite parameter set. Let *A*⊆*E*, *L* be a Lie algebra and

(22) $$\begin{array}{c} 𝔽{𝕊}_{A}\left(G\right)=\left\{\left(f,A\right)\in 𝔽𝕊𝕀\left(G,E\right)\;|\;F:A\to 𝔽\left(S\right)\right\} \end{array}$$

the set of bifuzzy soft sets over *L* and the parameter set *A*. It is trivial to verify that $\left(f,A)\;\tilde{\cup }\;(G,A),(f,A)\;\tilde{\cap }\;(g,A),(f,A)\cup (g,A),(f,A)\cap (g,A)\in 𝔽{𝕊}_{A}(G\right)$ for all (*f*, *A*), (*g*, *A*) ∈ *𝔽𝕊*_*A*_(*G*).

**Theorem 44**

Let (*f*, *A*) and (*g*, *A*) be (∈, ∈∨*q*)-bifuzzy soft Lie subalgebra over a Lie algebra *L*. Then so is (*f*, *A*)⊙(*g*, *A*).

**Theorem 45**

Let *L* be a Lie algebra with an identity *e*. Then (*𝔽* *𝕊𝕀*(*G*, *E*), ⊙, ∩) is a complete lattice under the relation ⊂.

## 5. Conclusions

Presently, science and technology are featured with complex processes and phenomena for which complete information is not always available. For such cases, mathematical models are developed to handle various types of systems containing elements of uncertainty. A large number of these models are based on an extension of the ordinary set theory such as bifuzzy sets and soft sets. In the current paper, we have presented the basic properties on bifuzzy soft Lie subalgebras. The most of these properties can be simply extended to bifuzzy soft Lie ideals. A Lie algebra is known algebraic structure and there are still many unsolved problems in it. In our opinion the future study of Lie algebras can be extended with the study of (i) roughness in Lie algebras and (ii) fuzzy rough Lie algebras.
